# Is *Strongyloides stercoralis *a risk factor for sepsis severity?

**DOI:** 10.1186/cc12916

**Published:** 2013-11-05

**Authors:** Patrícia Terra Alves, Marcelo Arantes Levenhagen, Fabiana de Almeida Araújo Santos, Omar Pereira de Almeida Neto, Liliane Barbosa da Silva Passos, Cezar Augusto dos Santos, Julia Maria Costa Cruz, Luiz Ricardo Goulart

**Affiliations:** 1 Genetics and Biochemistry Institute, Federal University of Uberlândia, Brazil; 2 Biomedical Sciences Institute, Federal University of Uberlândia, Brazil; 3 Clinical Hospital, Federal University of Uberlândia, Brazil

## Background

Sepsis is a complex disease with an initial proinflammatory profile triggered by an infection process, which is typically followed by a compensatory anti-inflammatory response, leading to immunosuppression. There are few cases in literature relating sepsis with opportunistic infections, such as strongyloidiasis, which may lead to severe clinical consequences due to hyperinfection. Human strongyloidiasis is a neglected tropical disease of major worldwide distribution, affecting millions of people. Despite of the fact that infection with *Strongyloides stercoralis *is usually self-limited and with low morbidity in immunocompetent individuals, it may become lethal in cases of immunosuppression, such as AIDS, corticosteroid treatment and transplantation. Our aim in this work was to investigate the presence of *S. stercoralis *antigens and anti-parasitic IgG in sepsis patients in a highly endemic area of strongyloidiasis.

## Materials and methods

Serum samples from 27 individuals with strongyloidiasis and 27 healthy subjects were used as positive and negative controls, respectively, according to their parasitological analyses. Additionally, 27 sepsis patients were also investigated. We have used ELISA tests to detect *S. stercoralis *antigens and IgG anti-*S. stercoralis *in all three groups. The cutoff value was determined by the ROC curves obtained by Prism 5.0 software.

## Results

IgG anti-*S. stercoralis *was detected in six patients; five under septic shock and one with sepsis. Among them, four were positive for the parasite antigen-antibody immune complex; three under septic shock and one with sepsis, demonstrating that 15% of sepsis patients were infected by the parasite, which may have significantly contributed with the hyperinfection presented by septic-shock patients (10%).

## Conclusions

There are only two reports of an association between *S. stercoralis *infection and immunosuppression, which led to lethal sepsis cases. However, our preliminary analysis through antigen-antibody immune complex demonstrated that this parasitic infection might be more common in sepsis than expected. The correct diagnosis of the causal infection in sepsis may support the correct therapeutic choice, which is fundamental to avoid the continuous spread of specific pathogen/parasite triggers that will eventually lead to hyperinfection, and consequently to severe sepsis.

